# The identification of the methylation patterns of tomato curly stunt virus in resistant and susceptible tomato lines

**DOI:** 10.3389/fpls.2023.1135442

**Published:** 2023-06-06

**Authors:** Precious Earldom Mulaudzi, Gerrit Koorsen, Imanu Mwaba, Nasima Banu Mahomed, Farhahna Allie

**Affiliations:** Molecular Plant-Pathology Laboratory, Department of Biochemistry, University of Johannesburg, Johannesburg, South Africa

**Keywords:** geminivirus, methylation, next-generation sequencing, plant-pathogen interactions, tomato

## Abstract

Tomato curly stunt virus (ToCSV) is a monopartite begomovirus infecting tomatoes in South Africa, with sequence similarity to tomato yellow leaf curl virus (TYLCV). While there are numerous reports on the mechanism of TYLCV resistance in tomato, the underlying mechanisms in the tomato-ToCSV pathosystem is still relatively unknown. The main aim of this study was to investigate and compare the global methylation profile of ToCSV in two near-isogenic tomato lines, one with a tolerant phenotype (T, NIL396) and one with a susceptible phenotype (S, NIL395). Bisulfite conversion and PCR amplification, coupled with a next-generation sequencing approach, were used to elucidate the global pattern of methylation of ToCSV cytosine residues in T and S leave tissue at 35 days post-infection (dpi). The extent of methylation was more pronounced in tolerant plants compared to susceptible plants in all sequence (CG, CHG and CHH) contexts, however, the overall methylation levels were relatively low (<3%). Notably, a significant interaction (p < 0.05) was observed between the viral genomic region and susceptible vs. tolerant status for CG methylated regions where it was observed that the 3’IR CG methylation was significantly (p < 0.05) higher than CG methylation of other genomic regions in tolerant and susceptible plants. Additionally, statistically significant (EdgeR p < 0.05) differentially methylated cytosines were located primarily in the genomic regions V2/V1 and C4/C1 of ToCSV. The relative expression, using RT-qPCR, was also employed in order to quantify the expression of various key methylation-related genes, *MET1, CMT2, KYP4/SUVH4, DML2, RDM1, AGO4* and *AGO6* in T vs. S plants at 35dpi. The differential expression between T and S was significant for *MET1, KYP4/SUVH4* and *RDM1* at p<0.05 which further supports more pronounced methylation observed in ToCSV from T plants vs. S plants. While this study provides new insights into the differences in methylation profiles of ToCSV in S vs. T tomato plants, further research is required to link tolerance and susceptibility to ToCSV.

## Introduction

Viruses of the family Geminiviridae are important plant pathogens of dicots and monocots globally and serve as one of the biggest threats to global food security and sustainability where the incidence and severity of the disease have increased over the last few decades (Boulton, 2005; Navas-Castillo et al., 2011; Promil Kapoor et al., 2019). These viruses possess either one (monopartite) and two (bipartite) small circular single-stranded genomes encapsidated in twinned-icosahedral particles (Navas-Castillo et al., 2011; Zerbini et al., 2017). Tomato curly stunt virus (ToCSV), in particular, contains a monopartite ssDNA genome, that contains six partially overlapping ORFs and is only the second begomovirus reported to infect *solanaceous* species in Southern Africa (Pietersen et al., 2000).

For host infection to occur, geminiviruses must successfully replicate and then move intracellularly, intercellularly, and systemically. Since geminiviruses have a relatively small genome, they have the ability to manipulate, reprogram and redirect the host’s cellular machinery in order to favour replication of their ssDNA genomes in the nucleus of host cells through a rolling-circle replication mechanism (Hanley-Bowdoin et al., 1999; Hanley-Bowdoin et al., 2004; Aguilar et al., 2020). Rolling circle replication involves the formation of double-stranded replicative form (RF) intermediaries that associate with cellular histone proteins to form minichromosomes (Pilartz and Jeske, 1992; Pilartz and Jeske, 2003) which then serve as the templates for the replication and transcription of viral genes. The altering of host gene expression results in severe physiological changes in infected plants and disease symptoms often manifest as leaf deformation, chlorosis, leaf curling, stunning etc.

Geminiviruses are known inducers of RNA silencing due to their bidirectional transcription and overlapping ORFs that results in the formation of dsDNA and dsRNA intermediates during rolling circle replication and transcription (Saunders et al, 1991; Jeske et al, 2002). It has been suggested that the initiation of virus-derived dsRNA originates from secondary folding structures in mRNA and/or overlapping transcripts of the virion sense and complementary sense ORFs of geminiviruses (Ding and Voinnet, 2007; Ramesh et al., 2017). The plant’s defense response to geminivirus invasion is mediated by the RNA-directed DNA methylation (RdDM) mechanism to suppress viral minichromosomes which silences the expression of viral genes by means of transcriptional gene silencing (TGS) (Zarreen and Chakraborty, 2020; Malavika et al., 2023).

RdDM and RNAi machinery are effective as a defense response as key role players involved in pathways are able to recognize viral RNAs and cleave them into small RNAs, which can then be used by both pathways to degrade viral RNA (RNAi) and silence viral DNA (Raja et al., 2014; Huang et al., 2016). RNA silencing pathways involve the generation of small RNA molecules, between 20-24nt long, which results in transcriptional gene silencing (TGS) or Post-transcriptional gene silencing (PTGS). The activation of RNA silencing and methylation as a defense mechanism in response to geminivirus infection has been reported in several studies. Methylation of the invading geminiviral genome is specifically mediated by the 24 nt class of siRNAs which forms part of the canonical RdDM pathway (Baulcombe, 2004; Ding and Voinnet, 2007; Raja et al., 2008; Buchmann et al., 2009; Raja et al., 2014). It functions as an antiviral defense mechanism that recruits a complex set of enzymes and proteins including domains rearranged methyltransferase (DRM2), AGONAUTE (AGO4), SAWADEE homeodomain homolog 1 (SHH1), polymerase IV and V, RNA-dependent RNA polymerase (RDR2) and DICER-like 3 (DCL3) (Pooggin, 2013). The DCL3 and AGO4 components specifically play a role in antiviral RdDM (Raja et al., 2014). Early studies have shown that the replication of Tomato golden mosaic virus was significantly reduced in tobacco (*Nicotiana tabacum*) protoplasts when their DNA was methylated *in vitro* (Brough et al., 1992). Similarly; cytosine methylation inhibits the replication of African cassava mosaic virus (Ermak et al., 1993). Additionally, Arabidopsis plants that were methylation-deficient, including cytosine methyltransferases, methyl-cycle enzymes, and Dicer-like proteins, were observed to be hypersusceptible to Cabbage leaf curl virus (CaLCuV) and Beet curly top virus (BCTV) (Raja et al., 2008).

Epigenetic modification such as DNA methylation involves three main mechanisms: DNA methylation, histone modifications, and RNA-mediated gene silencing. In plants, DNA methylation modification involves the transfer of -CH_3_ onto the C5 position of the cytosine to form 5-methylcytosine (5mCs) and it occurs in the sequence contexts: CG, CNG, and CHH (where H is A, T or C). This modification is catalyzed by the enzymes METHYLTRANSFERASE 1 (MET1), CHROMOMETHYLASE 3 (CMT3) and DOMAINS REARRANGED and METHYLTRANSFERASE 2 (DRM2), respectively (Law and Jacobsen, 2010; Zhang et al., 2018). There are numerous techniques that have been developed and utilized to investigate epigenetic marks at both genome-wide and sequence-specific levels, but bisulfite sequencing has become the gold-standard in mapping 5mC sites at single base-pair resolution (Frommer et al., 1992 and Clark et al., 1994). This technique is based on the treatment of denatured DNA with sodium bisulfite, which chemically deaminates unmethylated cytosine residues effectively converting unmethylated Cs to Ts (Frommer et al., 1992 and Clark et al., 1994). The use of bisulfite -sequencing to investigate the role of methylation in plant defense response to geminiviruses has already been extensively used and reported (Raja et al., 2008; Buchmann et al., 2009; Rodríguez-Negrete et al., 2013; Wang et al., 2018).

In this study, we aimed to investigate the global methylation patterns of ToCSV extracted from infected susceptible and tolerant leaf tissue at 35dpi, by using an amplicon next-generation sequencing approach. It was hypothesized that cytosine methylation patterns occur differently on the ToCSV genome in susceptible and tolerant tomato crops. These changes can affect the expression of ToCSV ORFs as well as plant genes involved in the defense response and may therefore contribute to tolerance mechanisms.

## Materials and methods

### Plant growth and agroinoculation

Two near-isogenic lines of tomato, tolerant (T, NIL396) and susceptible (S, NIL395), were obtained from Sakata (Lanseria, South Africa). The NILs were grown and maintained under a controlled environment in an insect-free chamber at 28 °C with a 16/8-h (light/dark) photoperiod. *Agrobacterium tumefaciens* C58C1 cultures were used for inoculation. The *Agrobacterium* containing ToCSV infectious viral clone (Accession: OK813888.1) were cultured in YEP media supplemented with 50 μg/ml of rifampicin and 50 μg/ml of kanamycin. Additionally, *Agrobacterium tumefaciens* C58C1 culture harboring an empty pCambia2300 plasmid, functioned as a negative control (mock-inoculated) containing 50 μg/ml of rifampicin when inoculated in YEP media. Both cultures were incubated at 30 ˚C for 48 hours at 200 rpm followed by co-cultivation into fresh YEP media supplemented with rifampicin (50 μg/ml) and kanamycin (50 μg/ml) until an OD_600_ of 0.8 was reached. *Agrobacterium* cultures were centrifuged at 8000 g for 5 mins followed by the removal of the supernatant. The pellet was then washed in sterile water followed by centrifugation at 8000 g for 5 min. Thereafter, the pellet was resuspended in YEP and used for the inoculation of tomato seedlings. The four-week-old tomato seedlings were wounded along the stem using a hypodermic needle and each seedling was inoculated with 100 µL of resuspended YEP media. For mock-inoculated plants, a volume of 100 µL of the *A. tumefaciens strain* C58C1 (pCambia2300) was inoculated in each seedling. The experiment was repeated three times independently, each consisting of four mock-inoculated plants and four ToCSV-inoculated plants.

### DNA extraction

Approximately 100 mg of the newly developed leaves below the apex were harvested in duplicates; for nucleic extraction. The leaf tissue was snap-frozen in liquid nitrogen and stored at -80 °C until needed. Total DNA was extracted from the harvested leaf tissue using the modified cetyltrimethylammonium bromide (CTAB) extraction method (Doyle and Doyle, 1987). Harvested snap-frozen leaf tissue was crushed to a fine powder using a micro pestle and resuspended in 500 µL of CTAB buffer (2% (w/v) CTAB, 1.4 M NaCl, 20 mM EDTA, 100 mM Tris-HCl (pH 8.0), 0.2% 2-mercaptoethanol, and 2% w/v PVP) and incubated at 65 °C for 60 min. For phase separation, 500µl of chloroform: isoamyl alcohol (24:1) was added, followed by centrifugation at 13 000 g for 10 min at room temperature. The aqueous phase was extracted to a new microfuge tube, and an equal amount of isopropanol was added to precipitate the DNA. The mixture was centrifuged at 13 000 g for 10 min, and the supernatant was removed. The pellet was washed twice in 1 ml of ice-cold 70% ethanol (v/v) followed by centrifugation at 13 000 g for 5 min. The pellet was air-dried and resuspended in TE buffer (1 M Tris, pH 8, and 0.5 M EDTA) supplemented with 200 µg/ml RNase A and stored overnight at 4°C. The extracted DNA was quantified using the NanoDrop™ 1000 Spectrophotometer (Thermo Fischer Scientific, Waltham, USA). The integrity of the DNA sample was assessed on a 1.0 % agarose gel stained with 1µg/ml ethidium bromide.

### Confirmation of ToCSV infection using conventional PCR

ToCSV infection was confirmed using universal degenerate begomovirus primers TY1 and TY2 (**Table S1**, Accotto et al., 2000), and conventional PCR using Dream Taq polymerase (Thermo Scientific, USA) according to the manufacturer’s protocol. The primer pair targets the coat protein (CP), producing a 580 bp amplicon (Accotto et al., 2000). The PCR reaction contained 1X dream Taq buffer, 0.2 mM of each primer, 0.025 U/µL Dream Taq polymerase, 0.2 mM deoxyribonucleotide triphosphate (dNTPs) and 500 ng/µl of DNA template. The PCR reaction was performed using Eppendorf master cycler (Merck, US) with the following conditions, initial denaturation at 95 °C for 2 min, followed by denaturation at 95 °C for 20 sec, annealing at 60 °C for 30 sec and extension at 72 °C for 40 sec for 35 cycles and, and a final extension at 72°C for 10 minutes. A volume of 5μL of PCR product was assessed on a 1 % agarose gel (w/v) stained with 1 µg/ml ethidium bromide.

### Real-time quantitative PCR of ToCSV titer

All qPCR assays were performed using Luna SYBR® Green fluorescence (New England Biolabs, USA), according to the manufacturer’s protocol and the CFX connect™ Real-Time System (Bio-Rad, USA). To determine the ToCSV viral titer in the T and S positively infected leaf tissue, the DNA samples were standardized to a final concentration of 10 ng/ul. Four biological samples were used for quantification for each of the plant lines and were performed in triplicate with a non-template control (NTC) of nuclease-free water for each run.

Each qPCR reaction was carried out in a final volume of 10 μL containing 5µl of the Luna universal qPCR master mix, 0.5 µl of forward and reverse primer (0.5µM), 2 µl DNA (2 ng/µL), and nuclease-free water. Two primer sets were used (**Table S1**), C2 primers that target the TrAP gene on ToCSV, and the 18S primers served as an internal loading control. Real-time PCR was performed for 40 cycles, with an initial denaturation at 95 °C for 60 sec followed by 40 cycles of denaturation at 95 °C for 15 sec and an extension of 60°C for 30 sec. A melt curve (60-95 °C) with continuous fluorescence measurement at the end of each amplification at a 0.05°C/s rate. Relative viral load was obtained using the 2^-ΔCt.^

### CpG island prediction and primer design

The complete sequence of the ToCSV viral construct was used to predict CpG islands using various software tools including: MethPrimer (Li & Dahiya, 2002), MethPrimer 2.0 (Li & Dahiya, 2002), Emboss (Madeira et al., 2019) and Sequence manipulation suite (Stothard, 2000). Default settings were maintained for all CpG island predictions. Bisulfite-specific primers (**Table S2**) were designed using Bisulfite Primer Seeker 12S (Zymo Research), to target the entire 2,767 bp of ToCSV (Accession no: OK813888.1)

### Bisulfite conversion and PCR

Total DNA was subjected to restriction digestion using the restriction endonuclease *BamH1* (Thermo Scientific, Waltham, USA). The endonuclease digestion was carried out in a reaction that contained 1X FastDigest ® buffer (Thermo Scientific, Waltham, USA), 1.25 U/µL *BamH1* enzyme and DNA template. The reactions were incubated at 37 °C for 16 hours and inactivated at 80 °C for 5 min. Followed by the addition of proteinase K (20mg/ml) and subsequent incubation for 15 min at room temperature and 10 min. Following the proteinase K treatment, the digested DNA was purified using the DNA clean-up (Zymo Research, Irvine, CA, USA) according to the manufacturer’s protocol and the concentration of the purified DNA was quantified using the NanoDrop™ 1000 Spectrophotometer (Thermo Fischer Scientific, Waltham, USA).

A total of 500 ng of purified DNA was used for bisulfite conversion, using the EZ DNA methylation Gold™ kit (Zymo Research, Irvine, CA, USA) according to the manufacturer’s protocol.

Bisulfite-modified DNA was used as a PCR template using Epimark Taq polymerase (New England Biolabs). PCR was carried out in a final volume of 20 µL, containing, 1X Epimark buffer, 200 µM dNTPs, 0.2 µM primer, 0.0125 U/µL PCR of the EpiMark Hot Start Taq DNA Polymerase and 100 ng DNA template. PCR for each primer set was performed independently using the Eppendorf master cycler (Merck, US) with the following cycling conditions; initial denaturation 95 °C for 30 sec, followed by 40 cycles of denaturation 95 °C for 30 sec, annealing at specific temperatures for each primer set (**Table S2**) for 60 sec, extension 68°C for 1 min and a final extension at 68°C for 5 min. To confirm positive amplification, 5µL of the PCR amplicons were separated on a 1 % agarose gel stained with 10 µg/mL ethidium bromide and visualized under ultraviolet light, using a ChemiDoc MP imaging system (Bio-Rad, USA).

#### Next-generation sequencing and data analysis

The twelve PCR amplicons, representing the complete ToCSV genome, were pooled together and sequenced at the Macrogen sequencing service (South Korea). A total of 3 biological replicates were sequenced per tomato line at 35dpi. The paired-end sequencing library was prepared by random fragmentation of the amplified DNA, after which 5’ and 3’ adapter ligation was performed. Adapter-ligated fragments were amplified by PCR and gel purified. Fragments were amplified into distinct, clonal clusters through bridge amplification. Templates were sequenced using Illumina SBS technology by Macrogen Inc. (South Korea) and standard quality control processes were followed. Raw sequence reads were filtered using Trimmomatic (Bolger et al., 2014) to remove low-quality sequences and sequencing adapters. Since amplicon sequencing was employed, duplicate reads were not removed. Processed reads were mapped to the reference ToCSV-genome (OK813888.1) using Bismark v0.23.1 (Krueger and Andrews, 2011), powered by the Bowtie2 aligner. Methylation bias was corrected by end-trimming reads (7-13 nt) as guided by M-bias plots. Methylated cytosines (in CG, CHG and CHH sequence contexts, respectively), were called using the Bismark methylation extractor. Methylation data were imported into SeqMonk v1.48.1 and cytosine methylation, or mean cytosine methylation over annotated genomic features, was quantified using the bisulfite sequencing quantitation pipeline. Sequence features with less than 10 mapped reads per feature, or for which only methylated or unmethylated calls were available, were not considered. Significant differentially methylated cytosines were identified using the proportion-based EdgeR statistical filter (p < 0.05 with multiple comparison correction) applied to the relevant replicate sets. Quantitative data were exported as SeqMonk feature reports and further data analysis and visualisation were performed using Tableau Public v2022.1.0. Two-way ANOVA and Tukey *post-hoc* tests were performed using the stats models library in Python v3.7.

### Total RNA extraction and cDNA synthesis

Total RNA was isolated from 100 mg tolerant and susceptible plant tissue at 35 dpi using the Direct-zol™ RNA miniprep kit (Zymo Research, USA) according to the manufacturer’s instructions. Thereafter, the quality and concentration of extracted RNA was determined using the NanoDrop® ND-1000 Spectrophotometer (Thermo-Fisher Scientific, USA). The integrity of RNA was confirmed by running a 1% agarose gel (w/v) stained with ethidium bromide (10mg/ml) alongside RiboRuler high-range RNA ladder (Thermo Scientific, USA).

Complementary DNA (cDNA) was prepared from 500 ng of total RNA using the Maxima H First Strand cDNA synthesis kit RT-qPCR (Thermo Scientific, USA) as per the manufacturer’s instructions. Freshly prepared cDNA was diluted with nuclease-free water to yield a concentration of approximately 10 ng/µl which was used as a template for subsequent qPCR gene expression studies.

### Relative gene expression of methylation-related genes using qPCR

Quantitative real-time PCR was performed using diluted cDNA at 35 dpi in both T and S lines. PCR reactions were set-up in a 10 µl volume containing 5 µl of Luna® qPCR SYBR (New England Biolabs, UK) master mix, 0.5 µl of gene-specific forward and reverse primers (0.5 µM) (**Table S3**), 10 ng of diluted cDNA and nuclease-free water. The Real-time PCR conditions included initial denaturation at 95˚C for 2 min, 40 cycles of denaturation at 95 ˚C for 15 s, and an annealing temperature specified per gene of interest for 30 s. The same reaction was set up separately to quantify actin as a reference gene to achieve relative gene expression. For each reaction, melting curve analysis was completed from 60-95˚C at a rate of 0.05˚C/s. Four biological replicates and 3 technical replicates, for ToCSV infected T and S leave tissue samples at 35 dpi were used along with a non-template control. The gene expression of each methylation-related gene was determined against Actin as an internal reference gene using relative quantification following the formula of 2^-ΔΔCt.^

## Results

As part of this study, a ToCSV infectivity study was conducted in T and S tomato lines at 15- and 35dpi. T (NIL 396) and S (NIL 395) tomato plants were grown and infected with ToCSV with the development of symptoms examined at 15- and 35dpi in a controlled growth room (Lapidot and Friedmann, 2002). The development of symptoms was first observed in S plants at 15 dpi where 75% of plants displayed minor foliar chlorosis and curling with the final stage showing 100% symptomatic infected plants with foliar chlorosis, cupping, and leaf reduction at 35 dpi (**Figure S1**). Additionally, the symptom severity of S-infected plants was more pronounced than that observed in T-infected plants at both 15 and 35dpi and T plants appeared almost symptomless throughout the study. Overall, while T plants all tested positive for ToCSV infection using conventional PCR, the display of symptoms on T plants was milder than those observed in the S plants. All T and S mock-inoculated plants did not display any disease symptoms over the 35-day period. Additionally, the relative viral load of ToCSV was measured in T and S plants and compared between 15dpi (onset of symptoms) and 35dpi (fully symptomatic) leaf tissue. Overall, S plants displayed a 2.43-fold increase in ToCSV titre between 15-and 35dpi compared to T plants (**Figure S1**).

**Figure 1:**
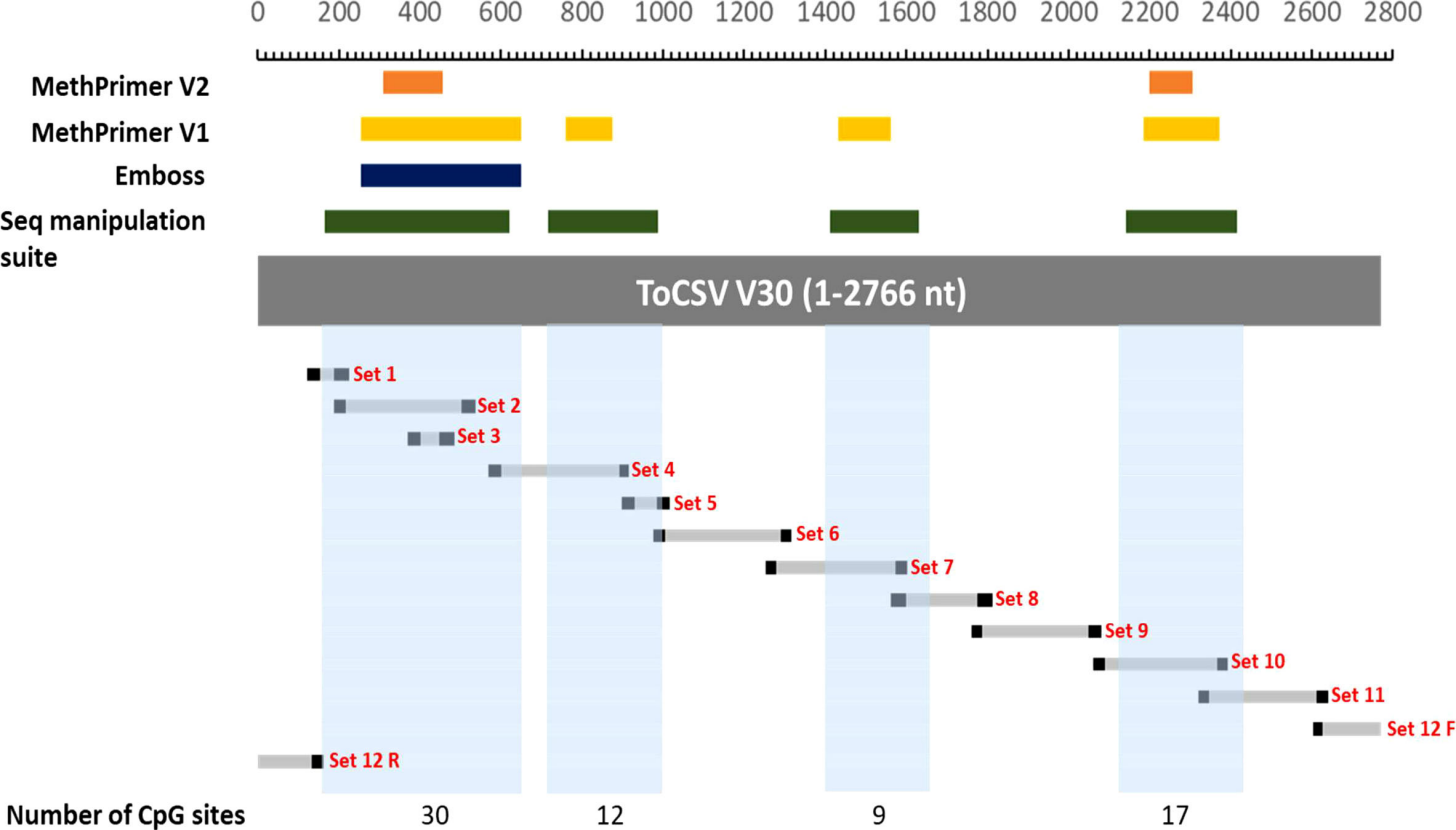
Mapping of CpG islands and methylation primers. The following coordinates were used to draw the map: MethPrimer2 CpG 1: 308-456; CpG 2: 2195-2306. MethPrimer1CpG1: 252-647; CpG 2: 757-872; CpG 3:1428-1558; CpG 4: 2182-2370. Emboss CpG1: 252-647. Sequence manipulation suite: CpG 1: 165-620; CpG 2: 714-986; CpG 3: 1410-1627; CpG 4: 2138-2411. Set 1 – 12 represent the amplification regions obtained using the different methylation primers. The blocks in light blue represent the overall CpG islands selected based on the regions identified by the different software programs used. The number of CpG sites indicated underneath are the numbers of consecutive CG nucleotides found in those regions.

For the ToCSV methylome study, the first investigation conducted was a bioinformatics one where four different software programs, namely, MethPrimer V1.0, MethPrimer V2.0, Emboss and Sequence manipulation suite were used to identify CpG islands present on the ToCSV genome. Based on the output from each program and a side-by-side comparison of the predicted CpG islands, a total of four CpG islands were identified on the ToCSV genome. CpG islands as indicated in **Figure 1**, where CpG island 1 is from nucleotide positions 165-647, CpG 2 island 2 at 714-986, CpG 3 at 1410-1627 and CpG 4 at 2138- 2411). The ToCSV genome contains a total of 580 cytosines which each serve as a potential target for methylation, however, CpG islands were hypothesized to be the primary “hotspots” for methylation.

**Figure 2:**
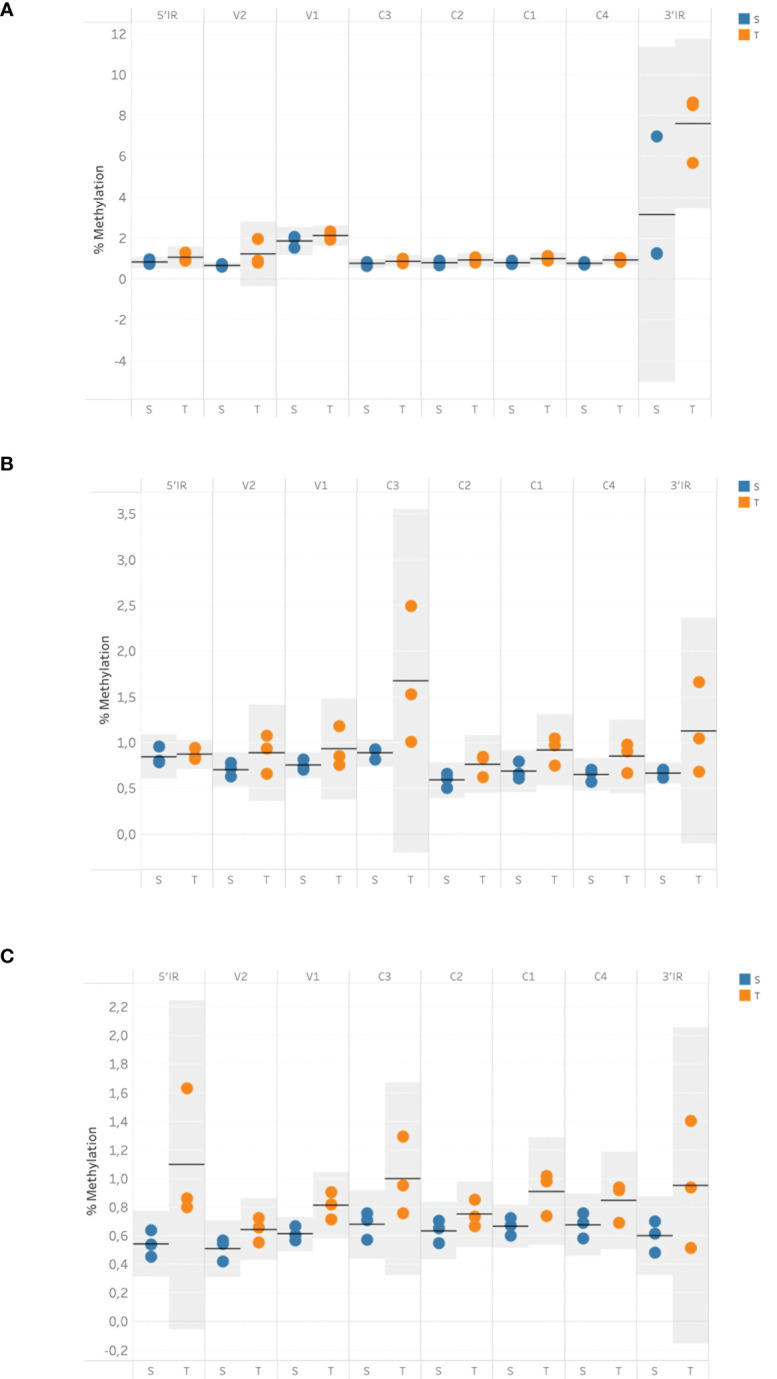
Mean cytosine methylation over viral genomic regions in tolerant and susceptible plants for CG **(A)**, CHH **(B)** and CHG **(C)** sequence contexts at 35 dpi. Methylation data were obtained using bisulfite sequencing and analyzed with the Bismark methylation extractor. The resulting data were imported into SeqMonk v1.48.1 and subjected to the bisulfite sequencing quantitation pipeline to quantify the mean cytosine methylation over annotated genomic features. Horizontal bars represent the mean value, and shaded areas represent the 95% confidence interval (CI) of the mean.

Twelve degenerate bisulfite-specific primer sets were then designed for amplicons ranging between 105-350nt that spanned the full genome of ToCSV (**Table S2**). Primers were designed to be degenerate to avoid sequence selection biases between unmethylated and methylated fragments during PCR. Twelve PCR fragments were successfully amplified and varied *via* visualization on 1% agarose TAE gels. For PCR amplicon NGS data, the samples yielded 28,667,890±2,956,855 pair-end reads on average, containing 11,940,400±5,574,831 reads mapped to the genome of ToCSV (OK813888.1; **Table S4**). Methylated cytosine calls resulted in an 8,823-fold to 50,711-fold coverage of viral genomic cytosine positions. The mean methylation levels over viral genomic regions were quantified for CG, CHG, and CHH sequence contexts (**Figure 2**). The coverage of reads mapped to the ToCSV offered a good high-resolution global landscape for ToCSV methylation in two different NIL of tomato plants. The extent of methylation was more pronounced in tolerant plants compared to susceptible plants in all sequence contexts. While the observed differences were small, differences were statistically significant (two-way ANOVA, p < 0.05, **Tables S5**-**S7**). Overall methylation levels were relatively low (<3%, **Figure 2**), with the exception of CG methylation of the 3’-IR region, which exhibited mean percentage methylation of 5.378% in virus isolated from susceptible and 7.577% isolated from tolerant plants, respectively (**Figure 2C**). These observations are consistent with previous reports of viral DNA methylation in infected tomato plants (Yang et al., 2011; Piedra-Aguilera et al., 2019).

Notably, a significant interaction (p <0.05) was observed between the viral genomic region and susceptible vs. tolerant status for CG methylated regions. Specifically, Tukey *post-hoc* tests revealed that 3’IR CpG methylation was significantly (p < 0.05) higher than CpG methylation of other genomic regions in tolerant and susceptible plants.

**Figure 3:**
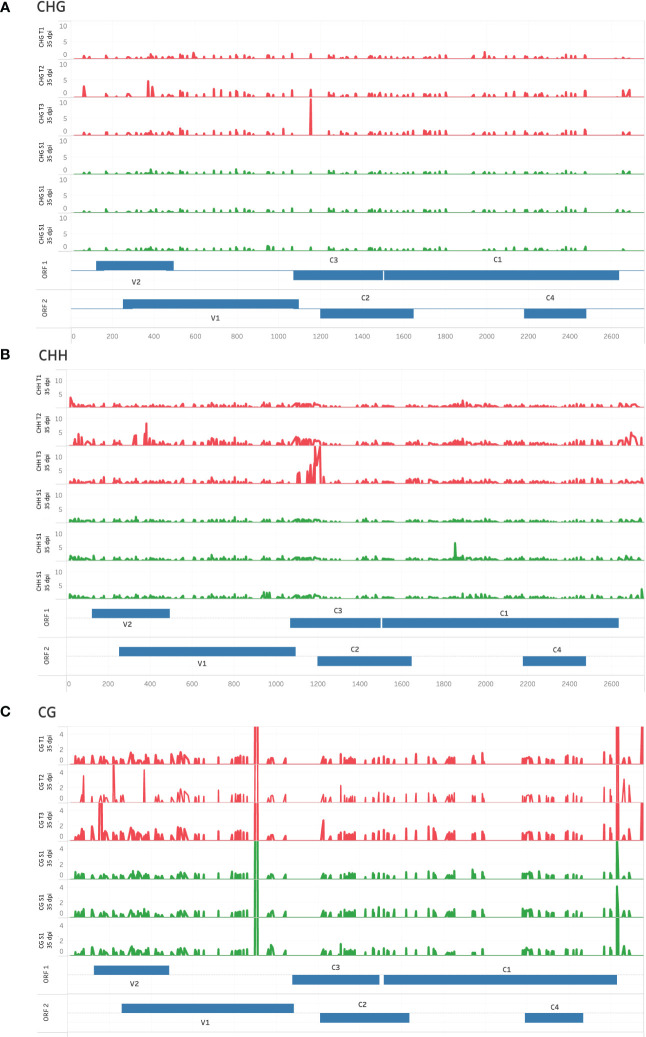
High-resolution cytosine methylation map of genomic regions in tolerant (T) and susceptible (S) ToCSV-infected tomato plants at 35 dpi. Percentage cytosine methylation of individual cytosines in CHG **(A)**, CHH **(B)**, and CG **(C)** sequence contexts are shown. Red traces represent tolerant lines and green traces susceptible lines. Genomic regions are annotated in two ‘ORF’ tracks, to distinguish between overlapping regions. ORF bars extending from the middle upwards reflect ORFs extending in the forward direction, while bars extending downward represent the reverse direction.

A dispersed pattern of low-level cytosine methylation was observed for viral DNA from T- and S-plants in CHG and CHH contexts, with sporadic spikes in methylation being more prevalent in T replicates compared to S (**Figures 3A, B**, respectively). In contrast, sharp CG methylation peaks were observed in the V1 and IR regions of the virus from tolerant and susceptible plants (**Figure 3C**). These observations differ from the cytosine methylation pattern observed for TYCLV-infected plants (Piedra-Aguilera et al., 2019), in which methylation peaked in the IR region of the virus.

**Figure 4:**
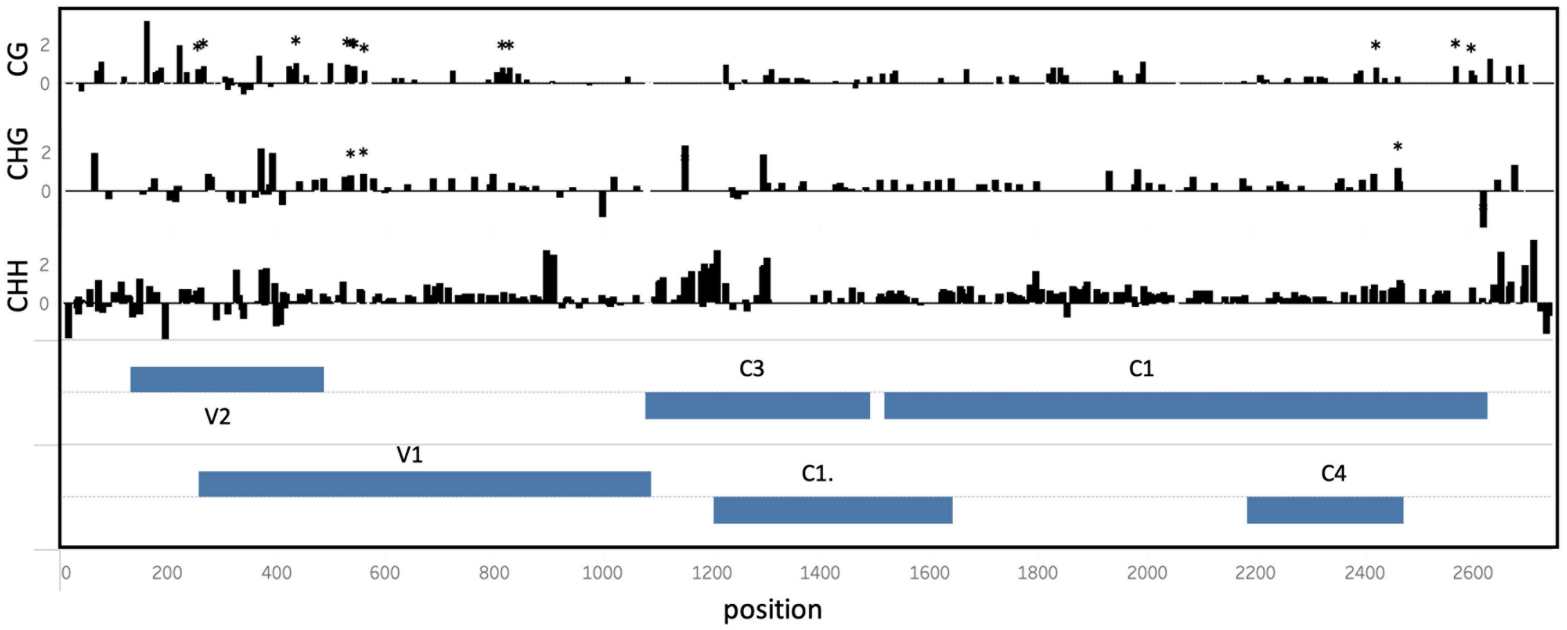
Differential cytosine methylation in the ToCSV genome isolated from tolerant (T) and susceptible (S). The log_2_-difference between cytosine methylation in T vs S plants is shown at different positions across the ToCSV genome, in CG, CHG and CHH sequence contexts. Genomic regions are annotated in two ‘ORF’ tracks. ORF bars extending from the middle upwards reflect genes extending in the forward direction, while bars extending downward represent the reverse direction. Asterisks indicate significantly differentially methylated cytosine positions (EdgeR, p < 0.05).

While differences in cytosine methylation occurred throughout the genome, statistically significant (EdgeR p < 0.05) differentially methylated cytosines were located primarily in the V2/V1 and C4/C1 viral genomic regions (**Figure 4**). V1/V2 and C1/C4 overlapping ORFs would result in the formation of double-stranded RNAs molecules triggering RNA silencing pathway. These regions were hypermethylated in viral genomes isolated from tolerant plants.

**Figure 5:**
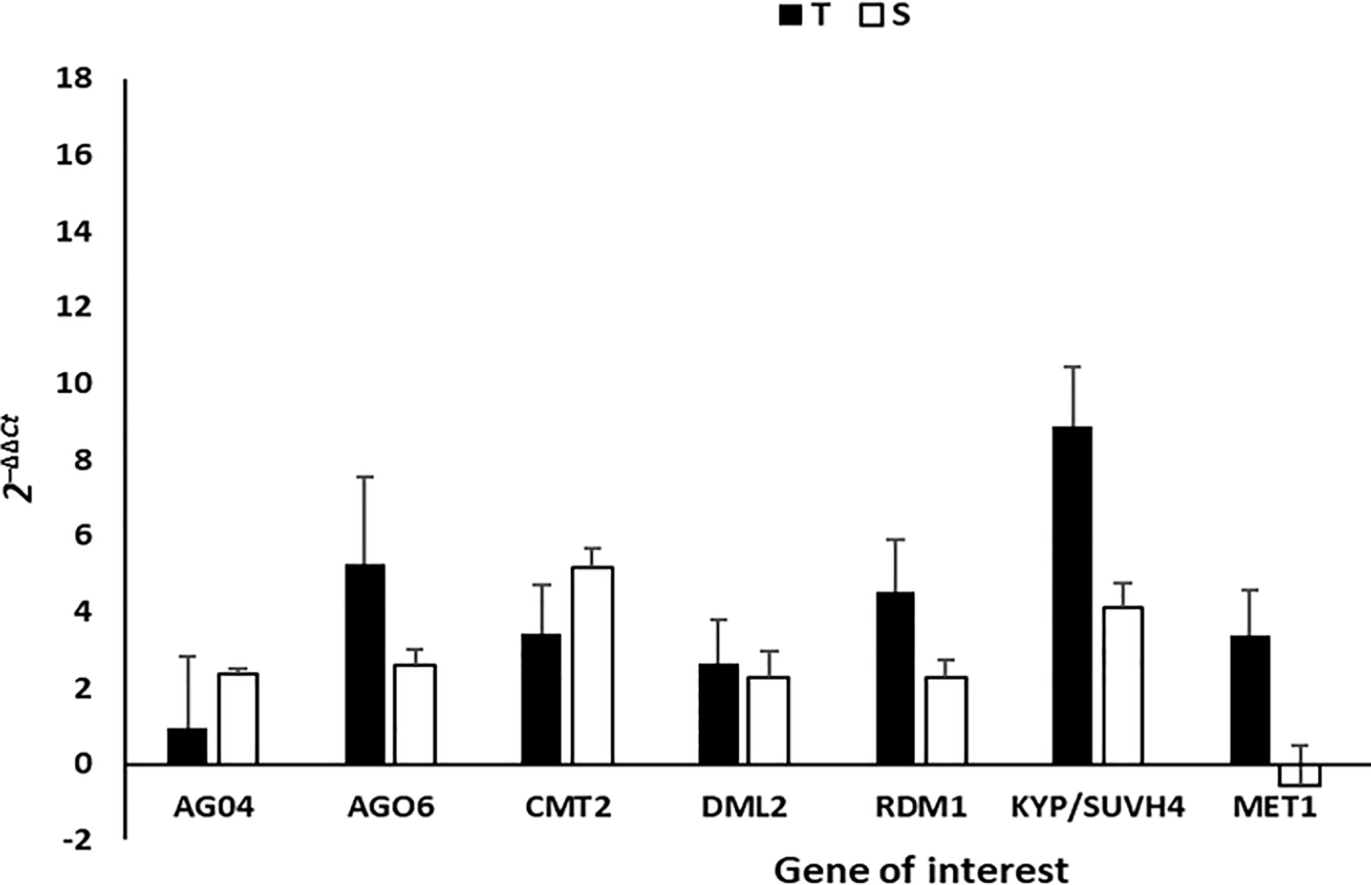
The gene expression (2^-ΔΔCt^) of methyltransferases, *CMT2, MET1; DML2, RDM1*, *AGO4*, *AGO6* and histone methyltransferase *KYP/SUVH4* was measured using RT-qPCR in T and S plants at 35 dpi, where Actin was used as the housekeeping gene. Expression values are representative of four biological replicates. The error bars illustrate the standard deviation of the mean with statistical significance determined using ANOVA and Student’s t-test, p<0.05. S = susceptible plant, T = tolerant plant.

In order to further investigate the methylation patterns observed from the NGS data, RT-qPCR was carried out on infected and mock-inoculated leave tissue in order to measure the transcript abundance of genes that encode enzymes and proteins that are known to be responsible for *de novo* and/or maintenance of cytosine methylation. These genes included: DNA METHYLTRANSFERASE (MET1), CHROMOMETHYLASE 2 (CMT2), KRYPTONITE (KYP4/SUVH4), DEMETER-LIKE protein (DML2), RNA-DIRECTED DNA METHYLATION1 (RDM1), ARGONAUTE4 (AGO4) and ARGONAUTE6 (AGO6). The relative expression of the seven genes measured, indicated that all genes were upregulated in T and S leave tissue at 35dpi, except *MET1*, which was found to be downregulated in S leave tissue at 35dpi (**Figure 5**). A Student’s t-test (p <0.05) carried out on the log_2_ 2^-ΔΔCt^ values showed that while all genes were upregulated in T and S plants, only *RDM1* (p =0.041), *KYP/SUVH4* (p=0.015), and *MET1* (p=0.006) were significantly higher in T plants compared to S plants. Furthermore, the relative expression for *RDM1* and *KYP/SUVH4* was two times higher in T vs. S plants and *MET1* was six times higher in T vs. S plants (**Figure 5**).

## Discussion

Investigating plant-pathogen interactions is important in elucidating and understanding the mechanisms governing plant disease. To date, the exact mechanisms underlying DNA methylation which controls plant immunity remain unclear and require further elucidation. The aim of this study was to use a PCR amplicon NGS sequencing approach to investigate the global methylation status of the ToCSV genome after infection in near-isogenic tolerant and susceptible tomato lines.

Traditionally, bisulfite sequencing is a method used to investigate methylation profiles by combining PCR amplification of the bisulfite-modified DNA with the subcloning of the amplicons into a plasmid followed by transformation into bacteria, plating on selective media and lastly Sanger sequencing of positively selected clones. For this study, cloning and subsequent steps have been eliminated and instead, the entire PCR population was sequenced using an NGS approach. To our knowledge, this is the first time an amplicon NGS approach has been used in order to determine the methylation status of a geminivirus, ToCSV. In a previous study by Paprotka et al. (2011) in 2011, a shotgun sequencing approach was employed in order to determine the methylation patterns of specifically circular DNA molecules of tomato yellow leaf curl Sardinia virus (TYLCSV) and Abutilon mosaic virus (AbMV) utilizing bisulfite treatment followed by rolling circle amplification. Our approach, however, did not restrict amplification to a specific confirmation of DNA and therefore PCR amplification of bisulfite-modified DNA was amplified from a heterogenous pool of DNA templates including geminivirus circular and linear DNAs as well as ssDNA and dsDNA.

CpG islands identified for ToCSV are indicated in **Figure 1**, CpG island 1 nucleotide position 165-647, CpG 2 island 2 714-986, CpG 3 1410-1627 and CpG 4, 2138- 2411. Interestingly, the patterns of methylation observed for this study are not restricted to the CpG islands 1-4 as hypothesized. The data presented here does however indicate that albeit low at <3%, that the ToCSV genome was methylated at 35dpi in both T and S plants, with the extent of methylation being more pronounced in tolerant plants compared to susceptible plants in all sequence contexts (CG, CHG and CHH). This is in line with observations made for in *Agrobacterium*-treated tomato (cv. Moneymaker) plants, where 2.5% methylation was observed on the TYLCV genome in two biological replicates.

It is also interesting that methylation was primarily observed for the 3’ end of the intergenic region (IR), V2/V1 and C4/C1 viral genomic regions. The IR contains the bidirectional promoter in begomovirus and contains the DNA binding sites for the viral replication protein (Rep, C1), host RNA polymerase II, and other key host factors required for virus replication. The IR of begomovirus genome is therefore vital to virus replication and viral gene transcription in plants. The IR has been proposed as the major target for host-induced methylation in previous studies as silencing of the promoter would prevent the downstream activation of replication *in planta.* A comparative study by Yadav and Chattopadhyay, also reports that bisulfite sequencing revealed a higher level of methylation in the IR of Mungbean yellow mosaic India virus (MYMIV) from a resistant variety of soybean compared to a susceptible variety.

The methylation pattern observed here for the C1/C4 region is similar to that observed in a study by Piedra-Aguilera et al in 2019, where two highly methylated regions located in the C1 ORF and around the IR were reported for TYLCV. Both these regions are crucial for viral replication and transcription and this might be one of the reasons that these genomic regions serve as the preferred targets by host defense systems. In geminiviruses, the replication‐associated protein (Rep), encoded by the C1 ORF, is expressed under the control of a bidirectional core promoter in the IR (Hanley-Bowdoin et al., 1999) and is essential for rolling circle replication (RCR). The C4 ORF is contained entirely within the C1 ORF, but in a different frame. While it has been shown to have a diverse number of functions, two of its primary designated functions include acting as an RNA silencing suppressor (Chellappan et al., 2004; Vanitharani et al., 2004; Ismayil et al., 2018) as well as acting as a pathogenicity determinant and contributing to successful and severe infection, and virus movement (Teng et al., 2010; Li et al., 2018; Li et al., 2020). Additionally, previous reports have indicated that a reduction in viral DNA titer and symptom development occurs when there is disruption of the C4 ORF of Tomato leaf curl virus (ToLCV, Rigden et al., 1994), Tomato yellow leaf curl virus (TYLCV, Jupin et al., 1994) and Beet curly top virus (BSCTV, Teng et al., 2010). The methylation observed in this study for the C1/C4 region is likely causing a disruption of C4 by TGS and would therefore reduce ToCSV viral infection and symptom development as is observed in the T lines and corresponds to other geminiviruses studies above.

There are currently six major resistance genes in tomato that are associated with resistance to TYLCV and have been described*, Ty-1* to *Ty-6*, and are being exploited in plant breeding. The first resistant gene against tomato yellow leaf curl virus (TYLCV) was reported to be *Ty-1* which encodes an RNA-dependent RNA polymerase that serves as an effective R-gene as it enhances transcriptional gene silencing (Butterbach et al., 2014). Plant breeders have introgressed some of these resistance genes identified in wild tomato cultivars into the domesticated tomato to reconstitute part of the resistance genes that were lost during domestication (Vidavski et al., 2008; Verlaan et al., 2013). These genes are derived from different wild-type tomato cultivars and were mapped using molecular markers. *Ty-1* (Zamir et al., 1994) is allelic with *Ty-3* (Ji et al., 2007) and was introgressed from *S. chilense* accession LA1969 and LA2779, respectively. The NIL lines used in this study were bred for the *Ty-1* R-gene (Sakata Seed Southern Africa) and we hypothesized that given the introgression of *Ty-1* in T-plants, it would have higher levels of methylated viral cytosines than S-plants. A study reporting a direct link between the presence of the *Ty-1* gene and an increase in cytosine methylation in geminiviruses was first reported by Butterbach et al. (2014) in 2014. It was reported that *Ty-1* results in hypermethylation at the promoter region of V1 (CP) in TYLCV collected from *Ty-1* tomato plants in comparison with susceptible tomato (Voorburg et al., 2021). A similar observation was made in our study where V1/V2 was also found to have higher levels of methylation in comparison to the S line. Since ToCSV is closely related to the Tomato yellow leaf curl virus (TYLCV) (Pietersen et al., 2008) and it was observed in this study that the tolerant NIL lines result in more pronounced methylation on the ToCSV genome, it stands to reason that TGS is enhanced in the tolerant line vs. the S line because of the presence of *Ty-1.*

Lastly, the final piece of evidence that we present there to support that methylation occurs more predominantly in the T line of tomato is the relative qPCR expression of key host DNA methyltransferases enzymes. The genes that showed significant (p <0.05) alteration in their expression in T and S tomato lines included *MET1, RDR1* and *KYP/SUVH4*. *MET1* primarily maintains CG methylation, whereas the *CMT2* methyltransferases are important for methylation at non-CG (CHH) sites (Bender, 2004). *KYP/SUVH4* is specifically identified as H3K9me2 methyltransferase and one of the argonaute proteins, *AGO4*, acts as a chaperone to target DNA thereby leading to methylation (Sun et al., 2015; He and Feng, et al., 2022). The change in gene expression of key methylation role players suggests that ToCSV is interfering with the proper functioning of the plant’s methylation cycle.

For S plants, *MET1* was the only gene shown to be downregulated in susceptible plants, in contrast, it was six times higher in T plants. Since MET1 is responsible for the maintenance of CpG DNA methylation, downregulation suggests that ToCSV may be suppressing the maintenance of CpG methylation in the S plants. The absence of methylation could be contributing to the promotion of viral replication and transcript which is in line with higher ToCSV titer observed in the S vs. T lines as well as the presence of severe symptoms at 15- and 35dpi (**Figure S1**).

KYP/SUVH4 is a histone H3 lysine 9 (H3K9) methyltransferase, which is generally a transcriptional repression mark (Krishnan et al., 2011), and is involved in chromatin modification of the viral genome. The induced expression of *KYP* is therefore indicative of histone methylation as T vs. S plants had two times higher *KYP* expression. We however cannot definitively report that induced *KYP* expression was solely targeting geminivirus minichromosomes and not also host plant chromatin as qPCR was carried out on total RNA (ToCSV and plant). However, based on a study by Luna et al. (2014) in 2014, KYP was implicated in the positive regulation and long-term priming in plant immune responses by promoting the phytohormone Salicylic acid which forms part of the systemic acquired resistance (SAR) pathway. If this ToCSV-tomato pathosystem is considered, then it can be proposed that the induction of KYP may not only directly be targeting viral minichromosomes but may also involve long-lasting up-regulation and/or priming of defense responses that enable a faster and/or stronger immune response in T plants.

RDM1 (RNA-DIRECTED DNA METHYLATION 1) is a small plant-specific protein required for RNA-directed DNA methylation (RdDM) (Sasaki et al., 2014). Similarly, to KYP expression, the expression of RDM1 was two times higher in T plants compared to S plants (**Figure 5**). RDM1 is required for de novo methylation of ssDNA at CHH sites and achieves its function by linking and interacting with Pol II and AGO4 (Sasaki et al., 2014). Since, RDM1 is required for RdRM, the higher expression in T plants could indicate rapid induction of RdDM pathways and hence TGS in T vs. S plants at 35dpi.

Methylation is a crucial epigenetic mechanism that regulates gene expression and can play a role in plant-virus interactions. Investigating methylation patterns on the ToCSV genome in tomato plants can have several implications for our understanding of the tomato-virus pathosystem. The findings of this study can be used for future studies in several ways. Firstly, it provides a new avenue for developing strategies for controlling geminivirus infections in crops. The study also highlights the importance of epigenetic mechanisms, such as DNA methylation, in the regulation of viral gene expression in plants. This could lead to further research into the role of epigenetics in plant-virus interactions and the identification of new targets or candidates for developing control strategies against ToCSV in South Africa.

In conclusion, we demonstrate for the first time the use of high-resolution PCR amplicon NGS sequencing to determine the global methylation status of the ToCSV in T and S tomato plants. While our results demonstrated that only a low percentage of ToCSV geminivirus DNA was methylated, the importance of methylation in plant defense cannot be dismissed, particularly in tolerant plants. Taken together, the lower viral titer, absence of symptoms, and more pronounced DNA methylation in T plants suggest a strengthening of transcription gene repression of ToCSV in T vs. S plants. The extent of methylation S plants may be delayed or not efficient enough to prevent active viral replication.

## Data availability statement

The original contributions presented in the study are publicly available. This data can be found here: https://www.ncbi.nlm.nih.gov/geo/query/acc.cgi?acc=GSE223671.

## Author contributions

FA and IM contributed to the conception and design of the study. PM conducted plant infections, bisulfite treatments, and PCR to generate a library used for next-generation sequencing. FA and NB conducted qPCR experiments and performed statistical analysis on qPCR data. GK conducted all bioinformatics and statistical analysis on NGS data. FA wrote the first draft of the manuscript. IM, PM, NB, and GK wrote sections of the manuscript. All authors contributed to the article and approved the submitted version.

## Supplementary material

Supplementary Figure 1
**(A)**: Symptoms on leaves of infected T and S plants at 35dpi. (a) Healthy/Mock tolerant plant. (b) ToCSV-infected T plant displaying mild curling. (c) Healthy/Mock susceptible plant. (d) ToCSV-infected S plant displaying severe foliar chlorosis and curling. (e) Height difference observed in S vs T infected plants. **(B)** Relative viral load quantification of ToCSV-inoculated T and S plants at 15- and 35 days post ToCSV inoculation. The quartiles relative to the median are represented as upper and lower boxes with the whiskers of each bar representing the standard deviation of the mean. Statistical significance was determined using ANOVA and Students t-test, p<0.05. N=3, black boxes represent tolerant plants and white boxes represent susceptible plants. * denotes statistical significance.
