# Supplementary figures and images for: The identification of the methylation patterns of tomato curly stunt virus in resistant and susceptible tomato lines
Source: Front Plant Sci. 2023 Jun 6;14:1135442. doi: 10.3389/fpls.2023.1135442 (PMC10281181; doi:10.3389/fpls.2023.1135442)

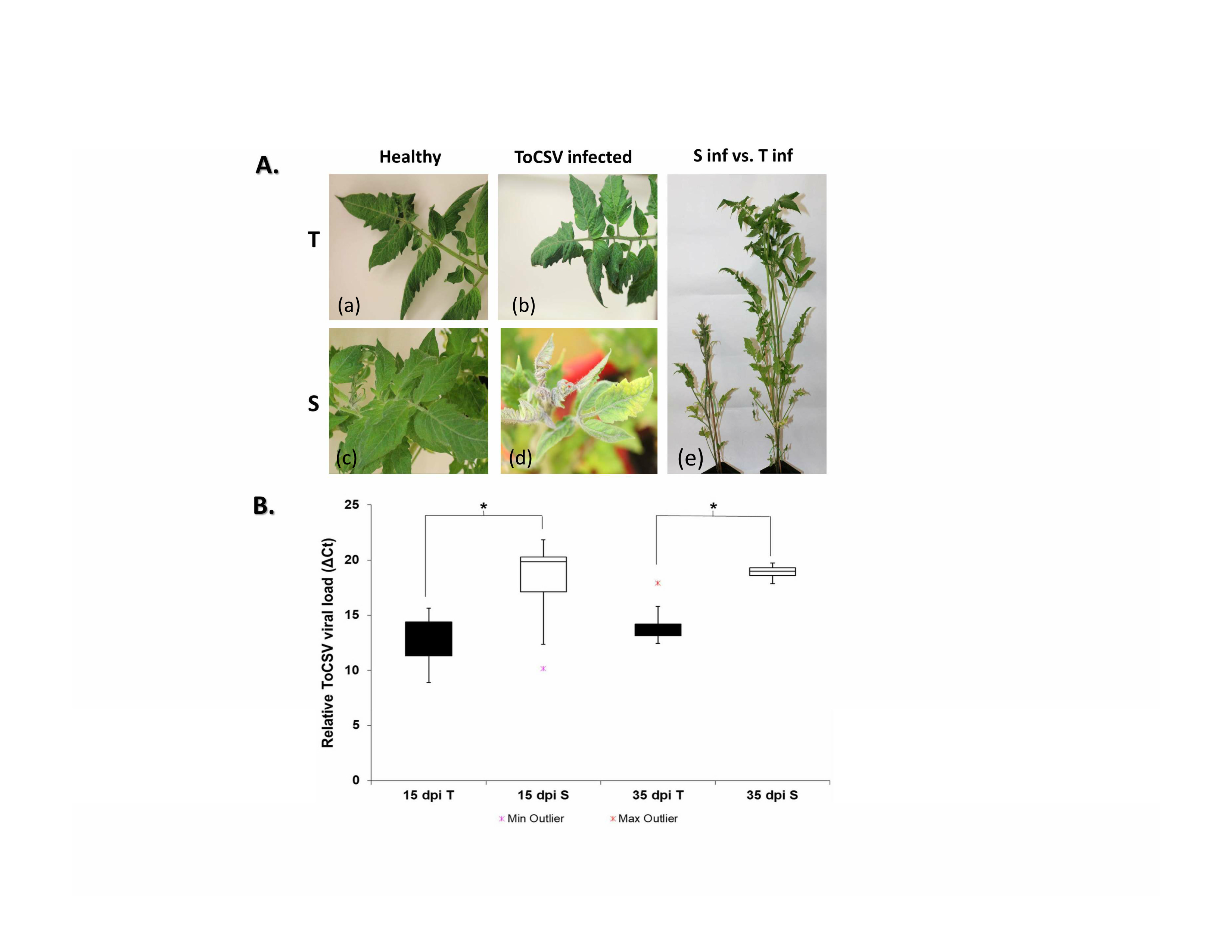

Supplement: Supplementary Figure 1 — (A): Symptoms on leaves of infected T and S plants at 35dpi. (a) Healthy/Mock tolerant plant. (b) ToCSV-infected T plant displaying mild curling. (c) Healthy/Mock susceptible plant. (d) ToCSV-infected S plant displaying severe foliar chlorosis and curling. (e) Height difference observed in S vs T infected plants. (B) Relative viral load quantification of ToCSV-inoculated T and S plants at 15- and 35 days post ToCSV inoculation. The quartiles relative to the median are represented as upper and lower boxes with the whiskers of each bar representing the standard deviation of the mean. Statistical significance was determined using ANOVA and Students t-test, p<0.05. N=3, black boxes represent tolerant plants and white boxes represent susceptible plants. * denotes statistical significance. [file Image_1.tif]
