# Supplementary material for: The identification of the methylation patterns of tomato curly stunt virus in resistant and susceptible tomato lines
Source: Front Plant Sci. 2023 Jun 6;14:1135442. doi: 10.3389/fpls.2023.1135442 (PMC10281181; doi:10.3389/fpls.2023.1135442)
Supplement: Supplementary file 2 [file Table_1.docx]

| Table S1: Primers for virus detection and quantification | | | |
| --- | --- | --- | --- |
| **Primer** | **5’-3’ sequence** | **T(A) °C** | **Amplicon (bp)** |
| TY1  TY2* | GCCCATGTA(T/C)CG(A/G)AAGCC- GG(A/G)TTAGA(A/G)GCATGC(A/C)GTAC | 60 | 580 |
| C2-F C2-R | ATCAAGGTCCAGCATCGAATAG CCTGATGAGCAGTGATGAGTT | 60 | 125 |
| 18S-F  18S-R | CGCGCGCTACACTGATGTATTCAA TACAAAGGGCAGGGACGTAGTCAA | 60 | 107 |
| A: annealing temperature  * Accotto et al. 2000 | |  |  |

***Supplementary Material***

***THE IDENTIFICATION OF THE METHYLATION PATTERNS OF TOMATO CURLY STUNT VIRUS IN RESISTANT AND SUSCEPTIBLE TOMATO LINES***
