# Supplementary material for: The identification of the methylation patterns of tomato curly stunt virus in resistant and susceptible tomato lines
Source: Front Plant Sci. 2023 Jun 6;14:1135442. doi: 10.3389/fpls.2023.1135442 (PMC10281181; doi:10.3389/fpls.2023.1135442)
Supplement: Supplementary file 3 [file Table_2.docx]

| **Table S2**: Bisulfite primers used to amplify ToCSV genome | | | | |
| --- | --- | --- | --- | --- |
| **Primer name** | **Position** | **Sequence** | **Number**  **of CpG islands** | **Annealing Temp.** |
| Meth 1 | 120-153  188-224 | 5ˈ-GGAAGTTATTATTGTTAAGATGTGGGATTTATTG-3ˈ  3ˈ-TCAACRAACTACAAATATTTAACAACAAACATACAAC-5ˈ | 6 | 50 |
| Meth 2 | 186-216  500-535 | 5ˈ-TYGTTGTATGTTTGTTGTTAAATATTTGTAG-3ˈ  3ˈ-TTCATACRACTAAATCTTACATAAACCTTCACAACC-5ˈ | 22 | 56.2 |
| Meth 3 | 368-401  447-484 | 5ˈ-TGATGTTTTTYGAGGTTGTGAAGGTTTATGTAAG-3ˈ  3ˈ-AAACCAAAACCCTTTATAATATCACTAATACACCTAAC-5ˈ | 5 | 50 |
| Meth 4 | 568-600  889-913 | 5ˈ-TAGGTGTATTAGTGATATTATAAAGGGTTTTGG-3ˈ  3ˈ-AAAAAAACRCTTCACAAAAACCTAC-5ˈ | 13 | 50 |
| Meth 5 | 897-929  982-1015 | 5ˈ-TTGTGAAGYGTTTTTTTAGGTTAAATAGTTATG-3ˈ  3-ˈAAAAACATAAATACATACCATATACAACAACAAC-5ˈ | 4 | 51.7 |
| Meth 6 | 975-1002  1288-1315 | 5ˈ-AGAATGYGTTGTTGTTGTATATGGTATG-3ˈ  3ˈ-CAACCTCCRAAAAAAACTAAAAATTCAC-5ˈ | 8 | 60.2 |
| Meth 7 | 1250-1276  1571-1599 | 5ˈ-TGTAAGGTYGTTTAGATTTGGAAGTTG-3ˈ  3ˈ-ACCACTATACTCAAATCCCAATCAAAATC-5ˈ | 16 | 56.2 |
| Meth 8 | 1560-1595  1773-1809 | 5ˈ-ATTYGATGTTGGATTTTGATTGGGATTTGAGTATAG-3ˈ  3ˈ-AAAACACTAAAAAAAATTCTTTAAAACCCAAAAAAAC-5ˈ | 6 | 60.2 |
| Meth 9 | 1757-1783  2077-2016 | 5ˈ-TYGTGTTGTTTTGTTAGTTTTTTTGGG-3ˈ  3ˈ-AAATAAAATTTTCCAAACTCCCCCAAAACC-5ˈ | 11 | 56.2 |
| Meth 10 | 2059-2087  2362-2390 | 5ˈ-GAAAAAGGAGAAATATAAGGTTTTGGGGG-3ˈ  3ˈ-TTCTTCRACCTAATATCCCCAAACAAATC-5ˈ | 14 | 60.2 |
| Meth 11 | 2316-2343  2608-2637 | 5ˈ-GTYGGAGTTGGATTTAGTTTTTTGAATG-3ˈ  3ˈ-AAATCAATCRATACCCATTAACCAAATAAC-5ˈ | 10 | 58.3 |
| Meth 12 | 2600-2623  133-162 | 5ˈ-TYGGAGGAGTTATTTGGTTAATGG-3ˈ  3ˈ-TTCRTTTAACAATAAATCCCACATCTTAAC-5ˈ | 14 | 60.2 |

***Supplementary Material***

***THE IDENTIFICATION OF THE METHYLATION PATTERNS OF TOMATO CURLY STUNT VIRUS IN RESISTANT AND SUSCEPTIBLE TOMATO LINES***
