# Supplementary material for: The identification of the methylation patterns of tomato curly stunt virus in resistant and susceptible tomato lines
Source: Front Plant Sci. 2023 Jun 6;14:1135442. doi: 10.3389/fpls.2023.1135442 (PMC10281181; doi:10.3389/fpls.2023.1135442)
Supplement: Supplementary file 5 [file Table_4.docx]

***Supplementary Material***

***THE IDENTIFICATION OF THE METHYLATION PATTERNS OF TOMATO CURLY STUNT VIRUS IN RESISTANT AND SUSCEPTIBLE TOMATO LINES***

**Table S4**. Sequence read statistics
